# Dexamethasone for adult patients with a symptomatic chronic subdural haematoma (Dex-CSDH) trial: study protocol for a randomised controlled trial

**DOI:** 10.1186/s13063-018-3050-4

**Published:** 2018-12-04

**Authors:** Angelos G. Kolias, Ellie Edlmann, Eric P. Thelin, Diederik Bulters, Patrick Holton, Nigel Suttner, Kevin Owusu-Agyemang, Yahia Z. Al-Tamimi, Daniel Gatt, Simon Thomson, Ian A. Anderson, Oliver Richards, Peter Whitfield, Monica Gherle, Karen Caldwell, Carol Davis-Wilkie, Silvia Tarantino, Garry Barton, Hani J. Marcus, Aswin Chari, Paul Brennan, Antonio Belli, Simon Bond, Carole Turner, Lynne Whitehead, Ian Wilkinson, Peter J. Hutchinson, British Neurosurgical Trainee Research Collaborative (BNTRC) and Dex-CSDH Trial Collaborators, Khaled Badran, Ian Coulter, Mathew J. Gallagher, Florence R. A. Hogg, Catherine Pringle, Adam Razak, Hamzah Soleiman, Rory Piper, Emma Toman, Marian Vintu, Adam Wahba, Anthony Wiggins, Kamal Makram Yakoub, Malik Zaben, Ardalan Zolnouria, Peter Bodkin, Emanuel Cirstea, Giles Critchley, Charlotte Eglinton, Louise Finlay, Daniela Georgieva, Nihal Gurusinghe, Nikolaos Haliasos, Damian Holliman, Kismet Hossain-Ibrahim, Masood Hussain, Jothy Kandasamy, Mary Kambafwile, Phillip Kane, Dipankar Nandi, Ravindra Nannapaneni, Laura Ortiz-Ruiz de Gordoa, Marios C. Papadopoulos, Dimitris Paraskevopoulos, Jash Patel, Manjunath Prasad, Nikolaos Tzerakis, Carol Brayne, Andrew Gardner, Andrew King, Kate Massey, Thais Minett, Patrick Mitchell, Phyo Myint, Elizabeth Warburton, Anthony Bell, Allison Hirst, Laurence Watkins, Peter McCabe, Martin Smith, Joan Grieve, Jonathan Cook

**Affiliations:** 1 Department of Clinical Neurosciences, University of Cambridge, Cambridge Biomedical Campus, Cambridge, CB2 0QQ UK; 2 Division of Neurosurgery, Addenbrooke’s Hospital, Box 167, Cambridge, CB2 0QQ UK; 3 Department of Clinical Neuroscience, Karolinska Institute, Stockholm, Sweden; 4 Wessex Neurological Centre, University Hospital Southampton NHS Foundation Trust, Tremona Rd, Southampton, Hampshire SO16 6YD UK; 5 Institute of Neurosciences, Queen Elizabeth University Hospital, 1345 Govan Road, Glasgow, UK; 6 Department of Neurosurgery, Sheffield Teaching Hospitals NHS Trust, Royal Hallamshire Hospital, Glossop Road, Sheffield, S10 2JF UK; 7 Department of Neurosurgery, Leeds General Infirmary, Great George Street, Leeds, LS1 3EX UK; 8 Southwest Neurosurgical Centre, Plymouth University Hospitals NHS trust, Plymouth, PL6 8DH UK; 9 Cambridge Clinical Trials Unit (CCTU), Coton House, Level 6, Cambridge Biomedical Campus, Box 401, Cambridge, CB2 0QQ UK; 10 Norwich Medical School, University of East Anglia, Norwich, NR4 7TJ UK; 11 Imperial College Healthcare NHS Trust, South Kensington Campus, London, SW7 2AZ UK; 12 Royal London Hospital, Barts Health NHS trust, Whitechapel Road, London, E1 1BB UK; 13 Department of Clinical Neurosciences, University of Edinburgh, Western General Hospitals NHS Trust, Crewe Road, Edinburgh, EH4 2XU UK; 14 NIHR Surgical Reconstruction and Microbiology Research Centre & University Hospitals Birmingham NHS Foundation Trust, School of Clinical and Experimental Medicine, University of Birmingham, Institute of Biomedical Research (West), Room WX 2.61, Edgbaston, Birmingham, B15 2TT UK; 15 MRC Biostatistics Unit, Robinson Way, Cambridge Biomedical Campus, Cambridge, CB2 0SR UK; 16 Clinical Trials Pharmacy, Addenbrooke’s Hospital, Cambridge Biomedical Campus, Cambridge, CB2 0QQ UK

**Keywords:** Chronic subdural haematoma, Dexamethasone, Neurosurgery, Neurology, Randomised control trial

## Abstract

**Background:**

Chronic subdural haematoma (CSDH) is a common neurosurgical condition, typically treated with surgical drainage of the haematoma. However, surgery is associated with mortality and morbidity, including up to 20% recurrence of the CSDH. Steroids, such as dexamethasone, have been identified as a potential therapy for reducing recurrence risk in surgically treated CSDHs. They have also been used as a conservative treatment option, thereby avoiding surgery altogether. The hypothesis of the Dex-CSDH trial is that a two-week course of dexamethasone in symptomatic patients with CSDH will lead to better functional outcome at six months. This is anticipated to occur through reduced number of hospital admissions and surgical interventions.

**Methods:**

Dex-CSDH is a UK multi-centre, double-blind randomised controlled trial of dexamethasone versus placebo for symptomatic adult patients diagnosed with CSDH. A sample size of 750 patients has been determined, including an initial internal pilot phase of 100 patients to confirm recruitment feasibility. Patients must be recruited within 72 h of admission to a neurosurgical unit and exclusions include patients already on steroids or with steroid contraindications, patients who have a cerebrospinal fluid shunt and those with a history of psychosis. The decision regarding surgical intervention will be made by the clinical team and patients can be included in the trial regardless of whether operative treatment is planned or has been performed. The primary outcome measure is the modified Rankin Scale (mRS) at six months. Secondary outcomes include the number of CSDH-related surgical interventions during follow-up, length of hospital stay, mRS at three months, EQ-5D at three and six months, adverse events, mortality and a health-economic analysis.

**Discussion:**

This multi-centre trial will provide high-quality evidence as to the effectiveness of dexamethasone in the treatment of CSDH. This has implications for patient morbidity and mortality as well as a potential economic impact on the overall health service burden from this condition.

**Trial registration:**

ISRCTN, ISRCTN80782810. Registered on 7 November 2014. EudraCT, 2014-004948-35. Registered on 20 March 2015.

Dex-CSDH trial protocol version 3, 27 Apr 2017.

This protocol was developed in accordance with the SPIRIT checklist. Available as a separate document on request.

**Electronic supplementary material:**

The online version of this article (10.1186/s13063-018-3050-4) contains supplementary material, which is available to authorized users.

## Background

Chronic subdural haematoma (CSDH) is an ‘old’ collection of blood and blood breakdown products in the subdural space. It is radiologically defined as a predominantly hypodense or isodense collection in the subdural space along the cerebral convexity on computed tomography (CT). It is especially common in older patients and in the UK, 5000 people aged > 65 years are diagnosed with a CSDH each year. It can happen following only a minor injury to the head or even in the absence of a known trauma [1]. Symptoms that can be attributed to a CSDH include headache, gait disturbance, falls, cognitive decline, focal neurological deficit, speech disturbance, decreased consciousness and seizures.

Patients with severe symptoms usually undergo an operation to evacuate the CSDH; while around 80% of patients recover well, around 10–20% experience recurrence of the CSDH requiring further surgery [1, 2]. Evidence from a previous CSDH trial looking at subdural drains demonstrated that a reduction in recurrence resulted in reduced mortality and rate of poor functional outcome at six months [1]. A considerable body of evidence suggests that administration of steroids could reduce CSDH recurrence and even the rate of primary surgical intervention [2–5]. This, in turn, might be expected to reduce mortality and morbidity and improve long-term functional outcome in patients with CSDH. While the mechanism of action of steroids in CSDH is not entirely understood, recent research suggests that inflammation may be responsible for driving the continued growth of CSDH [6–9]; therefore, steroids may help overcome this.

The Dex-CSDH trial (DEXamethasone in Chronic SubDural Haematoma) is a multi-centre, pragmatic, clinical phase III, randomised, double-blind, placebo-controlled trial of dexamethasone for up to two weeks in patients diagnosed with CSDH. Dexamethasone is one of the most potent synthetic analogues of the naturally occurring glucocorticoid hydrocortisone and has practically no water- and salt-retaining properties, so is suitable for use in patients with cardiac failure or hypertension [10]. The earliest application of steroids in neurosurgery was for patients with brain tumours and surrounding oedema, where 4 mg four times a day was established as the dose with maximum effect [11]. This dosing, with subsequent gradual weaning, continues to be used in neuro-oncology and a two-week course of dexamethasone was considered likely to provide the best balance in terms of clinical efficacy and risks in this study [12]. The dose and duration are also reflective of other studies in the field [13].

The potential impact of this trial is significant, as the results will determine whether steroids should be prescribed routinely for patients with symptomatic CSDH. If steroids are found to be effective, an impact on the speed of recovery and functional outcome of patients is expected. This will be measured by the primary outcome, the modified Rankin Scale (mRS) at six months. Additionally, this could reduce the rate of surgical interventions required, length of hospital stay, discharge destination and adverse events (AEs). As well as the impact on clinical outcome, there are health economic considerations that will be addressed by the trial.

### Trial rationale

We hypothesise that a two-week course of dexamethasone can improve the six-month functional outcome of patients with symptomatic CSDH by reducing the rate of CSDH-related surgical interventions and the recurrence rate.

### Trial objectives

#### Primary objective

To detect an 8% absolute difference in the rate of favourable outcome at six months between the two arms.

#### Secondary objectives

- Compare the long-term clinical effectiveness of dexamethasone versus placebo (six-month follow-up period)
- Compare the AEs and complications between the two arms
- Undertake a detailed economic evaluation between the two arms

#### Exploratory (mechanistic) objectives

- Assess the biological action of dexamethasone with CSDH fluid and blood analysis
- Assess the role of dexamethasone in cerebral perfusion and swelling in CSDH

## Methods

### Study setting

All study sites are in the UK. Patients are admitted to their local neurosurgical unit (NSU) following diagnosis of CSDH on CT. Local clinical neurosurgical teams review patients upon admission to the NSU and will assess eligibility for the Dex-CSDH trial. The decision for surgery or active monitoring is made on an individual patient basis by the admitting clinical team in conjunction with the patient and their families. This will not be affected by trial involvement, with both surgical and conservatively managed patients eligible for trial recruitment.

### Eligibility criteria

Screening of patients to determine eligibility for participation in the trial will be undertaken by the neurosurgical team upon admission to the NSU according to the following criteria.

#### Inclusion criteria

- Adult patients (aged ≥ 18 years)
- Symptomatic CSDH confirmed on cranial imaging (e.g. CT/magnetic resonance imaging [MRI]), predominantly hypodense or isodense crescentic collection along the cerebral convexity on CT
- Informed consent or Independent healthcare profession (IHP) authorisation

#### Exclusion criteria

- Patients with conditions where steroids are clearly contraindicated
- Patients who are on (or within one month of) regular oral or intravenous glucocorticoid steroids
- Previous enrolment in this trial for a prior episode
- Time interval from the time of the admission to the NSU to the first dose of the investigational medicinal product (IMP) > 72 h
- CSDH in the presence of a cerebrospinal fluid shunt
- Severe lactose intolerance or any known hypersensitivity to dexamethasone or other IMP excipients
- Patients with a previous history of psychotic disorders
- Unwillingness to take products containing gelatin
- Concurrent enrolment in any other trial of an IMP
- *Biochemical sub-study only*: active malignancy or currently receiving immunosuppressive drug therapy
- *MRI sub-study only*: renal dysfunction, pacemaker or metal implants

### Interventions

The trial aims to run in parallel to standard clinical care. The only difference between the trial pathway and the standard NHS pathway is the addition of a two-week tapering course of either dexamethasone or placebo (as per Table 1).

**Table 1:** Trial dosing regimen

Day	Capsules (n)	Equivalent dexamethasone dose
1, 2 and 3	4 in the morning, 4 at lunchtime	8 mg BD = 16 mg/day for 3 days
4, 5 and 6	3 in the morning, 3 at lunchtime	6 mg BD = 12 mg/day for 3 days
7, 8 and 9	2 in the morning, 2 at lunchtime	4 mg BD = 8 mg/day for 3 days
10, 11 and 12	1 in the morning, 1 at lunchtime	2 mg BD = 4 mg/day for 3 days
13 and 14	1 in the morning.	2 mg/day for 2 days
Total	62 capsules	124 mg over 14 days

Day 1 = day of first dose. Day 14 = last day of treatment. Day 1 treatment can be given as 1 combined dose of 16 mg (8 capsules) if needed, depending on the time of day the treatment is commenced

*BD* twice a day

The trial treatment can be delivered orally or by nasogastric tube. In special circumstances (such as patients who are nil by mouth for surgery), where study medication is missed at lunchtime, that day’s dose(s) may be taken later as long as it is on the same day. Otherwise, in the event of missing a dose of medication, these can be taken when remembered, but only up to the time of the next planned dose on the same day. No dose modifications are permitted within this trial. The trial is being carried out under a Clinical Trial Authorisation (CTA); for a list of known drug reactions and interaction with other therapies, see Appendix 1.

Irrespective of whether an operation is undertaken, patients will complete the two-week course of trial medication. Patients may be discharged or transferred to a local hospital before the completion of the two-week course; in this case, letters will be provided to the patient and medical and pharmacy teams at the local hospital along with the remaining trial medication to ensure that the course is completed. The exception to this will be in the event of a patient receiving study drug via the nasogastric route, where it will be stopped at discharge/ transfer if this is the case.

Trial teams will ensure compliance with treatment is documented, using source data which should include the inpatient medication administration record and the trial medication diary, as well as performing physical capsule counts during inpatient treatment where possible. Please refer to the Dex-CSDH IMP Handling Manual for further information.

Any concomitant therapy clinically required will be permitted, including gastroprotection as per local policy. A list of contraindicated concomitant therapies to be avoided during the trial is detailed in sections 4.3 and 4.5 of the current SmPC for dexamethasone [14]. Only concomitant therapies of interest will be recorded on the concomitant medication log in the case report form (CRF), including: gastroprotection; anti-diabetic medication; and single (intraoperative) dose of dexamethasone.

### Trial outcome measures

#### Primary outcome measure

mRS at six months after randomisation (Table 2). This scale was selected as it is a core instrument for measuring the degree of disability or dependence in daily activities of living and has previously been used in CSDH studies and stroke research, which affects a similar patient demographic [1, 15, 16].

**Table 2:** Modified Rankin Scale (mRS)

mRS score	Description
0	No symptoms at all
1	No significant disability despite symptoms; able to carry out all usual duties and activities
2	Slight disability; unable to carry out all previous activities, but able to look after own affairs without assistance
3	Moderate disability; requiring some help (e.g. with shopping/managing affairs) but able to walk without assistance
4	Moderately severe disability; unable to walk without assistance and unable to attend to own bodily needs without assistance
5	Severe disability; bedridden, incontinent and requiring constant nursing care and attention
6	Dead

#### Secondary outcome measures

1. Number of CSDH-related surgical interventions undertaken during the index admission
2. Number of CSDH-related surgical interventions undertaken during subsequent admissions in the follow-up period
3. Glasgow Coma Scale (GCS) at discharge from NSU and at six months
4. mRS score at discharge from NSU and at three months
5. Barthel Index at discharge from NSU, three months and six months
6. Mortality (at 30 days and six months)
7. EuroQOL (Quality of life) EQ-5D at discharge from NSU, three months and six months.
8. Length of stay in NSU
9. Discharge destination from NSU
10. Length of stay in secondary care
11. Health-economic analysis
12. AEs

#### Economic evaluation

An economic analysis will be conducted alongside the trial. Costs will be estimated from the viewpoint of the NHS and personal social services. Resources associated with provision of dexamethasone will thereby be monitored along with any surgical operation(s) to evacuate the CSDH, length of stay in NSU and any further hospital admissions /surgical procedures, e.g. for recurrence of the CSDH. Additionally, the level of informal care will also be monitored to estimate the opportunity cost for family, friends, carers and patients.

#### Exploratory (mechanistic) outcome measures

To assess the mechanism of action of dexamethasone, we will be collecting CSDH fluid and blood samples on selected patients who undergo surgery as part of their standard clinical care. Analysis of inflammatory biomarkers will be performed on blood and CSDH fluid and compared between the dexamethasone and placebo patients. Transcranial Doppler (TCD) and MRI may also be used in a sub-set of patients to measure cerebral blood flow patterns and assess whether this can be used to predict recovery and recurrence from CSDH.

### Participant timeline: trial assessments and schedule

**Fig. 1:**
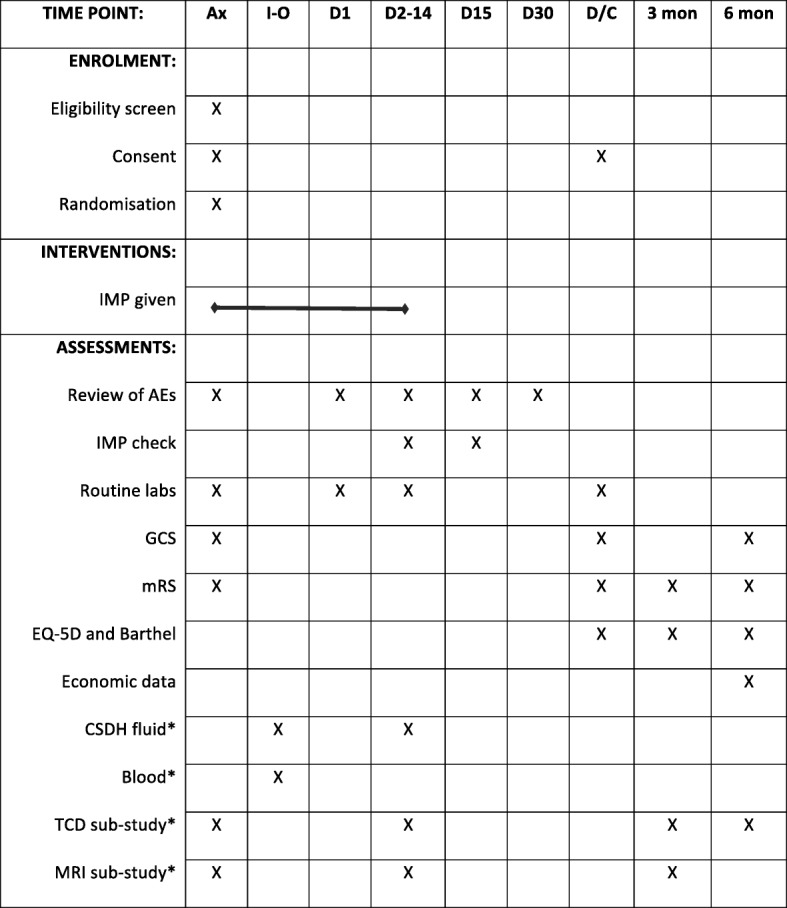
Schedule of assessments. * = only collected in patients recruited to sub-study in coordinating centre. Ax within 72 h of admission to NSU, AE adverse event, D day, D/C discharge (or death if sooner), EQ-5D European Quality of life-5 dimensions, IMP investigational medicinal product, I-O intraoperative, mon months, mRS modified Rankin Scale

All patients will have a medical history taken and a clinical examination as part of the routine standard of care, including: past medical history; injury-related events; neurological status; imaging (modality, date of examination and original images at selected sites); and routine lab results. Additional data will be collected on the exploratory outcomes (CSDH fluid, blood, TCD and MRI) if applicable, at the sponsor site only. Full details are available in the DEX-CSDH Laboratory and Imaging Manuals. Figure 1 shows a full schedule of trial assessments as per SPIRIT guidelines.

### Sample size

A sample size of 750 patients was determined with a power in the range of 81–92% and a two-sided significance of 5%, allowing for 15% missing data.

### Recruitment

The study commences with an internal pilot, stage 1 (feasibility study) to ensure 100 patients can be recruited by a limited number of centres within 12 months. Following successful completion of this, stage 2 (substantive study) will take place. The recruitment rate has been estimated at two patients per site per month. On the basis of Hospital Episode Statistics (HES) and data from the national CSDH audit [17], approximately 60–80 patients with a CSDH are admitted in a medium-sized NSU each year. Hence, the estimated recruitment rate is feasible. Patients will be monitored while in the acute NSU and followed up for a period of six months after recruitment.

### Treatment assignment, randomisation and blinding

Patients will be randomly assigned to either the control or intervention group with a 1:1 allocation as per a computer-generated randomisation schedule stratified by site using permuted blocks of random sizes. An interactive web-based response system (IWRS) will be used for allocating treatment packs to individual patients once confirmation that the inclusion criteria have been met has been confirmed.

Placebo is a capsule, visually indistinguishable from the active treatment and containing inactive excipients only. Dexamethasone capsules will consist of over-encapsulated dexamethasone 2 mg tablets. A proprietary brand will be used. The study drug will be supplied in individually numbered patient bottles. Capsules and packaging for both active and placebo arms will be identical in appearance at the point of issue to patients.

It is estimated that < 10% of eligible patients will have (or develop during the trial) swallowing difficulties, making oral IMP administration difficult or impossible/unsafe. In such cases, the blinded capsules may be opened at the point of administration by ward nursing staff and the contents dispersed in water, for administration either via oral route or a nasogastric tube. The administering nurse and potentially the trial patient will no longer be blinded, because the active dexamethasone is in tablet form that has been over-encapsulated and the placebo will be in powder form. To maintain blinding of the neurosurgeons, the presence of tablets being inside the opened capsule should not be documented in the medical notes.

Every effort must be made to maintain patient blinding when NG administration is used, by the patient not seeing the capsules being opened. Should, despite these efforts, the patient discover their treatment, they should be asked to not disclose their treatment allocation to any of the other medical personnel they interact with, e.g. surgeons, etc. The research staff and outcome assessors will remain blinded.

There are also clinical aspects that could potentially unblind trial team members to treatments allocated. Patients receiving dexamethasone will be more likely to have higher blood glucose levels compared to those receiving placebo. This may provide an indication but not proof that a patient is in the active arm. Concealment of glucose measurements will be difficult as clinical action may be required.

Any decision about surgery is made based on the severity of symptoms and/or progression of symptoms. Therefore, in cases where the IMP has been started before any neurosurgical intervention, a hint that the patient is in the active arm would have little influence on decisions about operative or non-operative management. Overall, we anticipate this occurring in such a small number of patients that the risk of bias is negligible and will not affect the overall findings of the study.

#### Emergency unblinding

Emergency unblinding will be managed according to the emergency unblinding procedure using the IWRS. Emergency unblinding requested by the patient’s clinical team will only occur in exceptional circumstances (e.g. need to treat a serious adverse event [SAE]) when knowledge of the actual treatment is essential for further management of the patient.

### Patient withdrawal

Each patient has the right to discontinue their participation in the trial at any time. If an unconscious patient regains capacity and makes a request to be withdrawn from the trial then this is accepted. Incapacitated patients may also be withdrawn from the trial if the consultee requests withdrawal. In addition, the investigator may withdraw the patient from their allocated treatment arm if, subsequent to randomisation, a clinical reason for not providing the drug treatment is discovered.

As the trial will be analysed on an intention to treat basis, any data collected will remain in the trial and the patient will continue to be followed up unless consent is withdrawn. Patients who have been withdrawn from the trial will not be replaced as the power calculation for the trial allows for a 15% loss to follow-up. All discontinuations and withdrawals will be documented. If a patient wishes to discontinue, anonymised data collected up until that point will be included in the analysis.

### Consent, enrolment and data collection

All patients who have been admitted to the NSU with a confirmed CSDH may be screened for eligibility. Screening will be carried out by a member of the clinical team and a log kept. Consent must be taken before study randomisation and study drug administration.

Where potential patients fulfil the eligibility criteria, they will be approached by a member of the research team who will provide the patient information sheet and clarify any information from the patient/relatives which may preclude recruitment. At Cambridge only, patients will also be screened for eligibility for the exploratory sub-studies. If they are eligible, they will be given an additional page in the patient information and consent sheet so that they can consider if they would like to take part in any of the additional sub-studies. If they do not wish to take part in these, it will not affect their recruitment to the main trial.

Wherever possible, informed consent will be obtained from the patient. However, due to the nature of the condition, this may not always be possible. If lacking capacity, patients with CSDH can still be enrolled in the trial if consent is obtained from:

i. the patient’s legal representative (if available in the hospital);
ii. IHP consent (if a patient’s legal representative is not available in the hospital) - this can be completed by someone who is not connected with the conduct of the trial, specifically:
  - the sponsor of the trial;
  - a person employed or engaged by or acting under the arrangements with the sponsor, and who undertakes activities connected with the management of the trial;
  - an investigator of the trial; or
  - a healthcare professional who is a member of the investigators’ team for the purposes of the trial.

Patients who regain capacity will be informed about the clinical trial and consent to continue will be sought during their in-patient stay and if still lacking capacity on discharge, at their six-month clinical follow-up appointment (if attended). If at any stage either the legal representative or the patient chose to withhold consent, then the patient will be withdrawn from the trial.

All enrolled patients will be offered an optional study wrist band, to be applied before their first dose of medication and worn while they are an inpatient. This highlights that they are taking part in a blinded study and helps the patient and nursing staff be aware of the study at all times and to reduce the risk of open label ward dexamethasone stocks being used in error.

Patients will be monitored as per routine clinical practice in the NSU until discharge and thereafter at approximately three and six months to assess clinical outcome. Follow-up will be by postal questionnaire. However, if after two weeks the questionnaire has not been returned, patients will be followed up by telephone. If after a further four weeks there is no response, then the patient will be deemed as lost to follow up. Where patients attend for a routine clinical follow-up, they will be reviewed by a blinded assessor.

### Data management

A final trial report will be written for publication and trial results will be presented internationally at meetings. All data will be entered into a CRF, which will be anonymised. The CRF will be accessible to trial coordinators, data managers, the investigators, Clinical Trial Monitors, Auditors and Inspectors as required. All CRF pages will be completed in a Good Clinical Practice (GCP)-compliant manner. All investigators and trial site staff involved in this trial must comply with UK Data Protection requirements and Trust Policy with regards to the collection, storage, processing and disclosure of personal information.

### Statistical methods

Analysis will be performed on an ‘intention-to-treat’ basis. The primary endpoint is the mRS at six months which is then dichotomised to favourable (0–3) versus unfavourable (4–6). The primary analysis will estimate the absolute difference between the two treatment arms in the proportions achieving a favourable outcome. A normal approximation will be used to produce 95% confidence interval and a two-sided *p* value testing the null hypothesis of no difference. Secondary analysis will include a proportional odds logistic regression of the mRS score adjusting for baseline covariates (age, GCS).

Assuming a favourable outcome rate of 80–85% in the control group, an 8% increase in the rate of favourable outcome (mRS 0–3) at six months is a plausible and clinically important treatment effect [1]. Using a two-sided test at the 5% significance level, a sample size of 750 patients (allowing for a 15% loss to follow-up) will enable us to detect this 8% absolute difference in the rate of favourable outcome with a power of 81–92%.

Further secondary endpoints will be summarised using appropriate techniques according to whether the variable is binary, categorical, continuous or time-to-event. Categorical and binary variables will be summarised using bar charts, frequency tables and comparisons made using logistic regression. Continuous variables will be summarised, broken down by treatment arm, using Box plots, mean, median, SD, max, min and compared using linear regression. Time-to-event variables will be summarised using Kaplan–Meier plots, and compared using the log-rank test.

#### Economic analysis

Appropriate unit costs will be assigned to each item of the aforementioned items of resource use (see trial outcome measures) using a standard price year. The mean incremental cost for those allocated to dexamethasone compared to placebo intervention over the six-month trial period will then be estimated, from both an NHS and personal social services perspective, and also with the addition of informal care costs. Assuming dominance does not occur (where one option is estimated to be more effective and less costly that the other option), the incremental cost-effectiveness ratio associated with dexamethasone will be estimated and assessed in relation to a range of cost-effectiveness thresholds, e.g. £20,000–30,000 per quality-adjusted life years (QALY) is recommended by National Institute for Health and Care Excellence (NICE) [18]. The associated level of uncertainty will also be characterised by estimating cost-effectiveness acceptability curves [19]. Additionally, sensitivity analysis will also be undertaken to assess the robustness of conclusions to change in key assumptions. In line with the outcome analysis, all analyses will initially be conducted on an intention-to-treat basis.

#### Interim analysis

An interim analysis, blinded to all except the study’s Independent Data Monitoring and Ethics Committee (IDMEC), will be performed after an appropriate number of patients have reached the six-month follow-up, to confirm the final sample size. The Trial Steering Committee (TSC), IDMEC and statistical team will agree jointly on the most appropriate timing of this interim analysis, taking into account the case mix and parameters the IDMEC wishes to estimate. If the sample size needs to be revised, we are able to incorporate the uncertainty in absolute favourable outcomes rates (80–85%) in order to achieve an acceptable conditional power as determined by the IDMEC. If sample size adjustment is necessary, the final analysis will adjust for the inflated type 1 error rate. The primary purpose of the internal pilot (first 100 patients) is to assess recruitment rates rather than to make sample size adjustments.

There are no defined criteria for the premature discontinuation of the trial. However, the IDMEC and TSC will make recommendations on the discontinuation of the trial following review of the ongoing patient safety and efficacy data presented at regular scheduled meetings. For the primary analysis, missing data will be assumed to be missing at random. A sensitivity analysis will be carried out by performing a complete case analysis. As the relevant covariates need to be recorded before the patient can be randomised, we aim to have minimal missing baseline data. There is also an excellent track record for UK-led neurosurgical studies in achieving extremely high rates for follow-up [20–22].

The end of the trial is the date that the last expected six-month follow-up questionnaire is completed for the last-recruited trial patient.

### Trial monitoring and safety

The TSC will provide overall supervision with respect to the conduct of the trial and be independently chaired by Professor Anthony Bell (St George’s, University of London, London, UK). The ethical and safety aspects of the trial will be overseen by an IDMEC, which will be chaired by Professor Martin Smith (The National Hospital for Neurology and Neurosurgery, London, UK).

The competent authority, the Medicines and Healthcare Products Regulatory Agency (MHRA), provided clinical trials authorisation before trial commencement. The protocol and trial conduct will comply with the Medicines for Human Use (Clinical Trials) Regulations 2004 and any relevant amendments. Development Safety Update Reports and Annual Safety Reports are submitted to the MHRA in accordance with UK requirements. It is the Chief Investigators responsibility to produce the annual reports as required.

Due to the patient demography and the clinical condition of CSDH, there may be many AEs throughout the initial admission. All patients are regularly monitored either in the intensive care environment or on the neurosurgical wards, but it is not practicable to record all AEs. Therefore, only AEs of special interest (AESIs) and SAEs will be reported. Some SAEs will be classified as ‘expected’ and therefore exempt from expedited reporting, although all will be recorded on a log (see Table 3). See Appendix 2 for a full list of AE descriptions and details.

**Table 3:** Adverse events of special interest (AESIs) and expected serious adverse events (ESAEs)

AESIs	ASAEs (non-reportable)
Metabolic	Perioperative
- Hyperglycaemia necessitating treatment or stopping of trial medication - New onset diabetes necessitating ongoing medical treatment at day 30 of follow-up - Hyperosmolar hyperglycaemic state	- Re-bleeding into cavity forming ASDH - Tension pneumocephalus - Intracerebral haemorrhage - Residual CSDH exerting mass effect - Seizures - Neurological worsening - Anaesthetic complications
Psychiatric	Early
- New onset psychosis	- Residual CSDH - Expansion of contralateral CSDH - Seizures
Gastric	Intermediate and Late
- Upper gastrointestinal side (e.g. heartburn, vomiting) - Peptic ulceration and gastro-intestinal bleeding	- Recollection of CSDH - Wound complications - Surgical site infection and subdural empyema - Epilepsy

*ASDH* acute subdural haematoma, *CSDH* chronic subdural haematoma

## Discussion

Despite the interest in, and potential impact of, conservative treatment options for CSDH patients, there currently exists no level 1 evidence to support any drug treatments. However, several studies have supported the use of dexamethasone and shown some evidence of its efficacy in reducing recurrence or as a primary treatment for CSDH [3–5, 9]. As a result, some clinicians are beginning to adopt dexamethasone as a treatment option in their routine practice. Assimilation of such new therapies into clinical care should be avoided until definitive evidence is available. The reasoning for this is exemplified by Prud’homme et al., who highlighted the potential adverse side effects associated with dexamethasone therapy in this patient population [23]. Such findings must be considered in trial design and it is evident that proving whether a medication is effective is not sufficient. One must also review the risk–benefit profile of a treatment to ensure the overall outcome affords significant benefit to the patient. Therefore, we have focused on functional outcome measures (e.g. mRS), so that the overall effect on quality of life, rather than change in imaging or tissue biomarker, is used to gauge success. This can only be achieved with a pragmatic, multi-centre trial, such as Dex-CSDH. Understanding the cost implications of new therapies is also important in NHS practice; therefore, health-economic analysis has also been incorporated in this trial protocol.

Potential limitations to this study include: reaching adequate recruitment in this patient population, as it has been highlighted that researchers can be reluctant to recruit elderly patients to randomised controlled trials [24]; and ensuring a range of severity of CSDH patients are included, as the more severely unwell patients will be unable to consent for themselves and NOK consent may be perceived as a barrier to recruitment if they are not immediately available. To help overcome this, IHP consent is an option and we hope this will be utilised to ensure broad inclusion of appropriate patients. Finally, as this study is limited to the UK, it may be questioned how applicable it is to other populations; however, we have sought to recruit centres covering a diverse range of patient demographics and therefore envisage the results will still be widely applicable.

The trial has successfully completed its feasibility phase of the first 100 patients and is now into the final phase of recruitment in 22 neurosurgical centres throughout the UK. Regular review of unblinded safety data is performed by the IDMEC, who have reported no concerns thus far. There is weekly oversight of the trial by a trial management group and biannual TSC meetings. Neurosurgical trainees have also been essential to the ongoing success of the trial through the British Neurosurgical Trainees Research Collaborative (BNTRC). This is a group that was founded in 2012 with the aim of encouraging high-quality multi-centre research within UK neurosurgery [25]. It promotes the structure where there is a trainee co-principle investigator (Co-PI) at each centre, helping oversee local site management and recruitment alongside the PI and research nurse team. The Dex-CSDH trial offers a model of how multi-centre trials can be successful in the UK with support from the wider neurosurgical community, including trainee collaboration.

## Trial status

Recruitment commenced on 13 August 2015 and is ongoing under protocol version 3 (27 Apr 2017) with 630 patients recruited as of 25 June 2018 across 22 UK sites. The protocol was written in line with the SPIRIT guidelines (see Additional file 1).

### Additional file

Additional file 1: SPIRIT checklist. (DOC 123 kb)

## Appendix 1

### Known drug reactions and interaction with other therapies

- **Hepatic microsomal enzyme inducers**: medicines that induce hepatic enzyme cytochrome P-450 isozyme 3A4 such as phenobarbital, phenytoin, rifampicin, rifabutin, carbamazepine, primidone and aminogluethimide may reduce the therapeutic efficacy of corticosteroids by increasing the rate of metabolism.
- **Hepatic microsomal enzyme inhibitors**: medicines that inhibit hepatic enzyme cytochrome P-450 isozyme 3A4 such as ketoconazole, ciclosporin or ritonavir may decrease glucocorticoid clearance. A reduction in corticosteroid dose may be needed to reduce the risk of adverse effects.
- **Antidiabetic agents:** corticosteroids may increase blood glucose levels. Patients may need dosage adjustment of any concurrent antidiabetic therapy.
- **Non-steroidal anti-inflammatory drugs (NSAIDs):** concomitant administration may increase the risk of gastrointestinal (GI) ulceration. Aspirin should be used cautiously in conjunction with corticosteroids in patients with hypothrombinaemia. The renal clearance of salicylates is increased by corticosteroids and steroid withdrawal may result in salicylate intoxication. Patients should be observed closely for adverse effects of either medicine.
- **Anticoagulants:** response to anticoagulants may be reduced or less often enhanced by corticosteroids. Close monitoring of the International Normalized Ratio (INR) or prothrombin time is recommended.
- **Antifungals:** the risk of hypokalaemia may be increased with amphotericin.
- **Cardiac glycosides**: there is a risk of toxicity if hypokalaemia occurs due to corticosteroid treatment.
- **Mifepristone**: the effect of corticosteroids may be reduced for 3–4 days after mifepristone.
- **Vaccines**: live vaccines should not be given to individuals with impaired immune responsiveness. The antibody response to other vaccines may be diminished.
- **Oestrogens**: oestrogens may potentiate the effects of glucocorticoids. The dose of corticosteroid may need to be adjusted if oestrogen therapy is commenced or stopped.
- **Somatropin**: the growth promoting effect may be inhibited.
- **Sympathomimetics**: there is an increased risk of hypokalaemia if high doses of corticosteroids are given with high doses of salbutamol, salmeterol, terbutaline or formoteral.
- **Diuretics**: excessive potassium loss may be experienced if glucocorticoids and potassium-depleting diuretics (such as frusemide and thiazides) or carbonic anhydrase inhibitors (such as acetazolamide) are given together.
- **Antacids**: concurrent use of antacids may decrease absorption of corticosteroids – efficacy may be decreased sufficiently to require dosage adjustments in patients receiving small doses of corticosteroids.

## Appendix 2

### Adverse event descriptions and details

Contains information regarding;

- AEs and their evaluation and reporting;
- adverse reactions (ARs);
- SAEs and reactions (SAR);
- Suspected unexpected serious adverse reaction (SUSAR);
- Reference Safety Information (RSI);
- Expected events;
- Evaluation and reporting of all AEs;
- Pregnancy reporting;
- Toxicity.

### Adverse event

Any untoward medical occurrence in a patient or clinical trial patient administered a medicinal product and which does not necessarily have a causal relationship with this treatment. An AE can therefore be any unfavourable and unintended sign (including an abnormal laboratory finding), symptom or disease temporally associated with the use of an investigational medicinal product, whether or not considered related to the investigational medicinal product.

Recording of AEs must start from the point of informed consent regardless of whether a patient has yet received a medicinal product.

### Adverse reaction to an investigational medicinal product (AR)

All untoward and unintended responses to an investigational medicinal product related to any dose administered. All AEs judged by either the reporting investigator or the sponsor as having a reasonable causal relationship to a medicinal product qualify as ARs. The expression reasonable causal relationship means to convey in general that there is evidence or argument to suggest a causal relationship.

### Serious adverse event or serious adverse reaction (SAE / SAR)

Any untoward medical occurrence or effect that:

- results in death;
- is life-threatening;
- requires hospitalisation or prolongation of an existing inpatients´ hospitalisation;
- results in persistent or significant disability or incapacity;
- is a congenital anomaly or birth defect;
- Is another important medical event.

Life-threatening in the definition of a SAE or SAR refers to an event in which the patient was at risk of death at the time of event; it does not refer to an event which hypothetically might have caused death if it were more severe. For the purposes of this trial, prolonged hospitalisation due to delayed transfer will not be considered a reportable SAE.

### Suspected unexpected serious adverse reaction (SUSAR)

A SAR, the nature and severity of which is not consistent with the information set out in the Reference Safety Information.

### Reference Safety Information

The information used for assessing whether an AR is expected is contained in the Summary of Product Characteristics (SmPC). For this trial the RSI is: section 4.8 – Undesirable effects, of the Aspen Pharma Trading Limited, Dexamethasone Tablets SmPC that has been approved by the MHRA for use in this trial.

### Expected events

- Expected AR /SARs

All expected ARs are listed in the latest version of the reference safety information as specified in the SmPC. This must be used when making a determination as to the expectedness of the AR. If the AR meets the criteria for seriousness it must be reported.

- Expected AE/SAEs

Expected procedural related AEs (if SAEs these are exempt from expedited reporting). Due to the nature of the condition and the characteristics of the patient population, affected individuals can often develop surgical and medical complications. In-hospital death can occur in approximately 5% of patients with a CSDH. AEs can be best classified in terms of perioperative, early, intermediate and late. The following AEs are ‘expected’.

#### Perioperative

Washout of the CSDH is normally performed through burr holes and therefore is not always under direct vision. This can lead to complications such as intracerebral haematoma (ICH), from inadvertent placement of a catheter during assisted washout or attempted division of membranes. It may also lead to incomplete washout, especially if membranes are still intact; therefore, ongoing CSDH and mass effect postoperatively. During washout, an acute source of bleeding may be agitated and if not recognised then a postoperative acute subdural haematoma can form in the cavity. The brain does not always fill the cavity immediately and therefore before closure the cavity is normally filled with saline to try and eliminate air. If a large amount of air becomes trapped in the cavity, it will lead to pneumocephalus which can be under tension and cause increased pressure and midline shift. Many of the patients undergoing this procedure are elderly and may have multiple co-morbidities; therefore, they are considered a high anaesthetic risk.

#### Early

Pneumocephalus can continue to be an issue in the first few days postoperatively. As the brain re-expands to fill the space, there is the additional risk of formation or increase in size of a contralateral CSDH. There is also a risk of seizures following evacuation of the CSDH which is more likely in patients with any perioperative complication such as ASDH, ICH and pneumocephalus.

#### Intermediate

Often a drain is placed initially to help with reduced risk of recurrence from CSDH; however, this is usually removed within 48 h. In the week following this, there is a risk that the CSDH can recollect. There is also a risk of infection, as with any surgical wound, and if significant then this could become a subdural empyema if there is also a recollection of subdural fluid. Poor wound healing or dehiscence is a risk, particularly as the patients are mostly elderly and will have thin skin which does not heal rapidly. There is an ongoing risk of developing epilepsy in the first few weeks postoperatively.

#### Late

The biggest risk of the longer term is of recollection of the CSDH which may require further surgical treatment. There is also an ongoing risk of developing late epilepsy.

### Evaluation of adverse events

The Sponsor expects that AEs are recorded from the point of informed consent regardless of whether a patient has yet received a medicinal product. Individual AEs should be evaluated by the investigator. This includes the evaluation of its seriousness, causality and any relationship between the investigational medicinal product(s) and/or concomitant therapy and the adverse event. AEs should only be recorded for the duration of the patient’s hospital stay.

i. Assessing causality

Definitely: a causal relationship is clinically/biologically certain. This is therefore an AR.

Probable: A causal relationship is clinically / biologically highly plausible and there is a plausible time sequence between onset of the AE and administration of the investigational medicinal product and there is a reasonable response on withdrawal. This is therefore an AR.

Possible: a causal relationship is clinically / biologically plausible and there is a plausible time sequence between onset of the AE and administration of the investigational medicinal product. This is therefore an AR.

Unlikely: a causal relation is improbable; another documented cause of the AE is most plausible. This is therefore an AR.

Unrelated: A causal relationship can be definitely excluded; another documented cause of the AE is most plausible. This is therefore an AE.

Unlikely and Unrelated causalities are considered NOT to be trial drug-related.

Definitely, Probable and Possible causalities are considered to be trial drug-related. A pre-existing condition must not be recorded as an AE or reported as an SAE unless the condition worsens during the trial and meets the criteria for reporting or recording in the appropriate section of the CRF.

ii. Assessing severity

Mild: the patient is aware of the event or symptom, but the event or symptom is easily tolerated.

Moderate: the patient experiences sufficient discomfort to interfere with or reduce his or her usual level of activity.

Severe: significant impairment of functioning; the patient is unable to carry out usual activities and / or the patient’s life is at risk from the event.

iii. Recording of adverse events

This clinical trial is being conducted in a critical emergency condition. It is important to consider the natural history of the critical medical event affecting each patient enrolled, the expected complications of this event and the relevance of the complications to the procedures.

All AESIs, including expected systemic and procedure-related AEs, will be assessed by the Investigator and recorded in detail in the medical notes and CRFs. Results of locally performed clinical laboratory tests (full blood count, coagulation, biochemical markers) will also be recorded in the CRF.

AESIs recorded during the trial will be sent to the coordinating centre. At the conclusion of the trial, all AESIs will be subject to statistical analysis, and the analysis and subsequent conclusions will be included in the final trial report. AESIs will be review at TSC meetings. SAEs and SARs must be reported to the Sponsor.

### Reporting adverse events

i. SAEs

Each Principal Investigator must report all reportable SAEs to the Chief Investigator, via the Trial Coordinating Centre, using the trial-specific SAE form, within 24 h of their awareness of the event. (For details of SAEs that are exempt from expedited reporting requirements, please see Section 12.3.) The Chief Investigator is responsible for ensuring the assessment of all SAEs for expectedness and relatedness is completed and the onward notification of all SAEs to the Sponsor immediately but not > 24 h of first notification. The sponsor has to keep detailed records of all SAEs reported to them by the trial team.

The Chief Investigator is also responsible for prompt reporting of all SAE findings to the competent authority in each member stage (e.g. the MHRA) if they could:

- adversely affect the health of patients;
- impact on the conduct of the trial;
- alter the risk-to-benefit ratio of the trial;
- alter the competent authority’s authorisation to continue the trial in accordance with Directive 2001/20/EC.

The completed SAE form can be faxed or emailed. Details of where to report the SAEs can be found on the Dex-CSDH SAE form and page 2 of the protocol.

ii. Suspected unexpected serious adverse reactions (SUSARs)

All suspected adverse reactions related to an investigational medicinal product (the tested IMP and comparators) which occur in the concerned trial, and that are both unexpected and serious (SUSARs) are patient to expedited reporting.

The Sponsor delegates the responsibility of notification of SUSARs to the Chief Investigator. The Chief Investigator must report all the relevant safety information previously described, to the:

- Sponsor;
- competent authorities in the member state (e.g. the MHRA);
- Ethics Committee in the concerned member states.

The Chief Investigator shall inform all investigators concerned of relevant information about SUSARs that could adversely affect the safety of patients.

#### Fatal or life-threatening SUSARs

All parties listed in above must be notified as soon as possible but no later than seven calendar days after the trial team and Sponsor has first knowledge of the minimum criteria for expedited reporting. In each case, relevant follow-up information should be sought and a report completed as soon as possible. It should be communicated to all parties within an additional eight calendar days.

#### Non-fatal and non-life-threatening SUSARs

All other SUSARs and safety issues must be reported to all parties listed above as soon as possible but no later than 15 calendar days after first knowledge of the minimum criteria for expedited reporting. Further relevant follow-up information should be given as soon as possible.

#### Minimum criteria for initial expedited reporting of SUSARs

Information on the final description and evaluation of an AR report may not be available within the required time frames for reporting. For regulatory purposes, initial expedited reports should be submitted within the time limits as soon as the minimum following criteria are met:

- a suspected investigational medicinal product;
- an identifiable patient (e.g. trial patient code number);
- an AE assessed as serious and unexpected, and for which there is a reasonable suspected causal relationship;
- an identifiable reporting source, and, when available and applicable:
- a unique clinical trial identification (EudraCT number or in case of non-European Community trial’s the sponsor’s protocol code number);
- a unique case identification (i.e. sponsor’s case identification number).

#### Follow-up reports of SUSARs

In case of incomplete information at the time of initial reporting, all the appropriate information for an adequate analysis of causality should be actively sought from the reporter or other available sources. Further available relevant information should be reported as follow-up reports. In certain cases, it may be appropriate to conduct follow-up of the long-term outcome of a particular reaction.

#### Format of the SUSARs reports

Electronic reporting is the expected method for expedited reporting of SUSARs to the competent authority. The format and content as defined by the competent authority should be adhered to.

### Pregnancy reporting

All pregnancies within the trial should be reported to the Chief Investigator and the Sponsor using the relevant Pregnancy Reporting Form within 24 h of notification. Pregnancy reporting would stop three months after the patient’s last dose of IMP for example.

Pregnancy is not considered an AE unless a negative or consequential outcome is recorded for the mother or child/fetus. If the outcome meets the serious criteria, this would be considered a SAE.

### Toxicity: emergency procedures

In the event of suspected toxicity, the trial drug will be withdrawn. In the event of emergency unblinding, this will be managed by the appropriate SOP.

## Abbreviations

AE: Adverse event
AESI: Adverse event of special interest
CRF: Case report form
CSDH: Chronic subdural haematoma
CT: Computed tomography
Dex: Dexamethasone
EQ-5D: EuroQol Quality of Life questionnaire
GCP: Good Clinical Practice
GCS: Glasgow Coma Scale
HES: Hospital Episode Statistics
IDMEC: Independent Data Monitoring and Ethics Committee
IHP: Independent healthcare professional
IMP: Investigational medicinal product
IWRS: Interactive web-based response system
MHRA: Medicines and Healthcare products Regulatory Agency
MRI: Magnetic resonance imaging
mRS: Modified Rankin Scale
NG: Nasogastric
NSU: Neurosurgical unit
SAE: Serious adverse event
TCD: Transcranial Doppler
TSC: Trial Steering Committee
