# Correction to: Dexamethasone for adult patients with a symptomatic chronic subdural haematoma (Dex-CSDH) trial: study protocol for a randomised controlled trial

**DOI:** 10.1186/s13063-019-3283-x

**Published:** 2019-03-18

**Authors:** Angelos G. Kolias, Ellie Edlmann, Eric P. Thelin, Diederik Bulters, Patrick Holton, Nigel Suttner, Kevin Owusu-Agyemang, Yahia Z. Al-Tamimi, Daniel Gatt, Simon Thomson, Ian A. Anderson, Oliver Richards, Peter Whitfield, Monica Gherle, Karen Caldwell, Carol Davis-Wilkie, Silvia Tarantino, Garry Barton, Hani J. Marcus, Aswin Chari, Paul Brennan, Antonio Belli, Simon Bond, Carole Turner, Lynne Whitehead, Ian Wilkinson, Peter J. Hutchinson, British Neurosurgical Trainee Research Collaborative (BNTRC) and Dex-CSDH Trial Collaborators

**Affiliations:** 1 Department of Clinical Neurosciences, University of Cambridge, Cambridge, Biomedical Campus, Cambridge, CB2 0QQ UK; 2 Division of Neurosurgery, Addenbrooke’s Hospital, Box 167, Cambridge, CB2 0QQ UK; 3 Department of Clinical Neuroscience, Karolinska Institute, Stockholm, Sweden; 4 Wessex Neurological Centre, University Hospital Southampton NHS Foundation Trust, Tremona Rd, Southampton, Hampshire, SO16 6YD UK; 5 Institute of Neurosciences, Queen Elizabeth University Hospital, 1345 Govan Road, Glasgow, UK; 6 Department of Neurosurgery, Sheffield Teaching Hospitals NHS Trust, Royal Hallamshire Hospital, Glossop Road, Sheffield, S10 2JF UK; 7 Department of Neurosurgery, Leeds General Infirmary, Great George Street, Leeds, LS1 3EX UK; 8 Southwest Neurosurgical Centre, Plymouth University Hospitals NHS trust, Plymouth, PL6 8DH UK; 9 Cambridge Clinical Trials Unit (CCTU), Coton House, Level 6, Cambridge Biomedical Campus, Box 401, Cambridge, CB2 0QQ UK; 10 Norwich Medical School, University of East Anglia, Norwich, NR4 7TJ UK; 11 Imperial College Healthcare NHS Trust, South Kensington Campus, London, SW7 2AZ UK; 12 Royal London Hospital, Barts Health NHS trust, Whitechapel Road, London, E1 1BB UK; 13 Department of Clinical Neurosciences, University of Edinburgh, Western General Hospitals NHS Trust, Crewe Road, Edinburgh, EH4 2XU UK; 14 NIHR Surgical Reconstruction and Microbiology Research Centre & University Hospitals Birmingham NHS Foundation Trust, School of Clinical and Experimental Medicine, University of Birmingham, Institute of Biomedical Research (West), Room WX 2.61, Edgbaston, Birmingham, B15 2TT UK; 15 MRC Biostatistics Unit, Robinson Way, Cambridge Biomedical Campus, Cambridge, CB2 0SR UK; 16 Clinical Trials Pharmacy, Addenbrooke’s Hospital, Cambridge Biomedical Campus, Cambridge, CB2 0QQ UK

**Correction to: Trials (2018) 19:670**

**https://doi.org/10.1186/s13063-018-3050-4**

After publication of the original article [1], the authors notified that that one of the BNTRC institutional collaborator names was misspelled. Both incorrect and correct spellings are presented below.

- Originally published name

Ardalan Zolnouria

- Correct name spelling

Ardalan Zolnourian
